# When priapism reveals Chronic Myeloid Leukemia: A case report

**DOI:** 10.1016/j.eucr.2026.103496

**Published:** 2026-05-29

**Authors:** Muhammad Farras Afif Syamhudi, Agung Prasetyo Nitisasmito, Encep Ivan Setiawan

**Affiliations:** a Faculty of Medicine, Lampung University, 35141, Bandar Lampung, Indonesia; b Urology Division, Dr. H. Abdul Moeloek Regional Hospital, 35112, Bandar Lampung, Indonesia; c Urology Resident, Department of Urology, Faculty of Medicine, Gadjah Mada University, 55281, Sleman, Indonesia

**Keywords:** Priapism, Chronic myeloid leukemia, Distal shunt, Ebbehoj technique

## Abstract

Priapism is a rare complication associated with Chronic Myeloid Leukemia (CML). We present the case of a 20-year-old man who experienced a painful erection lasting for 10 hours. Laboratory tests indicated extreme leukocytosis. Initial treatments, including aspiration and intracavernosal therapy, were unsuccessful, leading to the need for an emergency distal shunt, which effectively relieved the condition. Further investigations confirmed the diagnosis of CML. The patient responded well to cytoreductive therapy and remained free of recurrence during follow-up.

## 1 Introduction

Priapism is a urological emergency defined as a persistent penile erection lasting more than 4 h without sexual stimulation.^1^ This condition is time sensitive; if not adequately managed, it can increase the risk of irreversible ischemic injury and subsequent erectile dysfunction.^1^^,^^2^ The primary goal of therapy is rapid detumescence of penile flaccidity through stepwise interventions, including corporal aspiration, intracavernous sympathomimetic injection, and surgical shunting.^3^^,^^4^ Prompt management is crucial, as delayed detumescence can lead to poor functional outcomes.^1^

Ischemic priapism, the most common type, is usually caused by idiopathic causes, pharmacological agents, or hemoglobinopathies.^5^ However, in some patients, it can be a symptom of a hematologic malignancy.^6^ The underlying pathophysiology involves hyperviscosity and leukostasis, leading to impaired cavernosal outflow.^7^^,^^8^

Priapism occurs in less than 5% of leukemia cases, making it an uncommon symptom in CML.^6^ This rarity presents a unique challenge for urologists, especially when standard first-line treatments are ineffective.^7^ In these situations, it is crucial to balance the timely escalation of surgical intervention with the need for immediate hematologic assessment and cytoreductive therapy.^6^^,^^9^

In this report, we present a case of priapism in a young adult man with extreme hyperleukocytosis who underwent distal shunt placement due to failure of intracavernosal therapy, leading to a newly recognized diagnosis of CML. This case highlights the importance of awareness of rare etiologies in urologic emergencies.

## 2 Case presentation

A 20-year-old male with no prior medical history presented to the emergency department with a persistent penile erection lasting approximately 10 hours without sexual stimulation. The condition was associated with progressively worsening pain, with a visual analog scale (VAS) score of 9/10. The patient denied any history of trauma, medication use, penile discoloration, or sensory changes. Urinary function was normal.

**Fig. 1:**
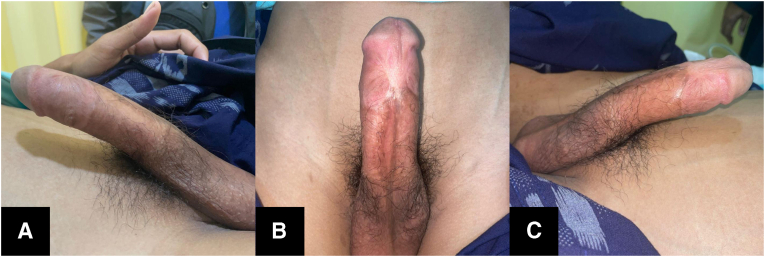
Clinical image of the penis in a state of maximum erection. (A) Right lateral view of the penis. (B) Ventral view of the penis. (C) Left lateral view of the penis.

On physical examination, vital signs were within normal limits. The patient appeared mildly pale, with conjunctival pallor noted. Abdominal examination revealed hepatomegaly and splenomegaly. Genital examination demonstrated a fully erect penis (Fig. 1). Laboratory findings revealed anemia (hemoglobin 8.6 g/dL), a normal platelet count (236,000/μL), and extreme leukocytosis (538,440/μL). Based on the clinical findings, a diagnosis of ischemic priapism secondary to a hematologic malignancy was suspected.

Initial management with corporal aspiration and intracavernosal phenylephrine injection was performed but failed to achieve detumescence. Consequently, an emergency distal shunt procedure using the Ebbehoj technique was undertaken. The procedure was performed under local anesthesia, followed by multiple percutaneous punctures using a No. 11 scalpel blade through the glans penis into the corpora cavernosa. A compression dressing was subsequently applied to the glans penis.

Supportive management included intravenous fluids, analgesics, antibiotics, and placement of two intravenous lines. A Foley catheter was inserted to monitor the patient's urine output. Packed red cells were prepared for transfusion. Intraoperatively, an estimated blood loss of approximately 200 mL of dark blood was noted. The patient received 4 units of normal saline and 400 mL of packed red cells (PRC).

**Fig. 2:**
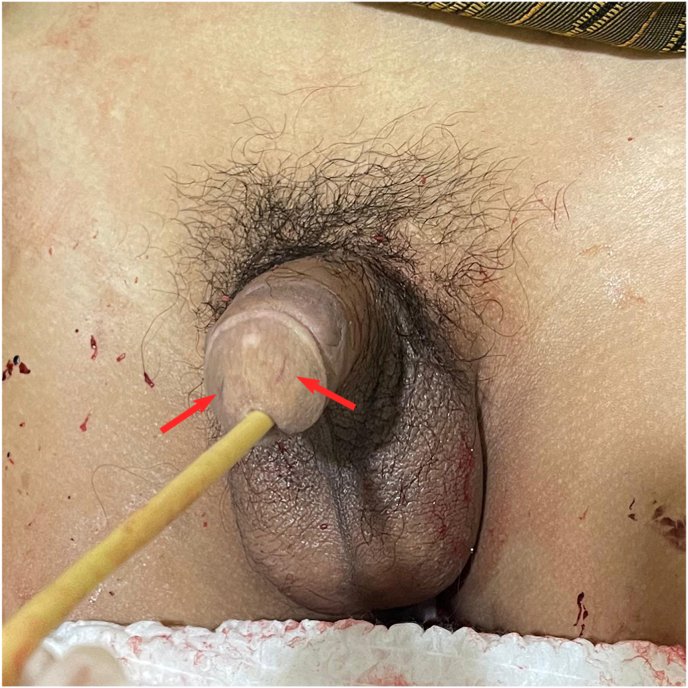
Distal shunt location with ebbehoj technique was performed.

**Fig. 3:**
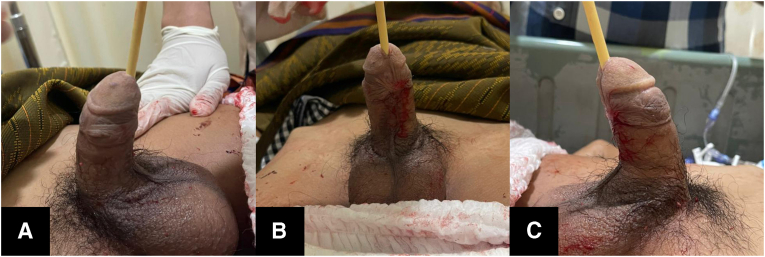
Clinical image of the penis 3 hours after distal shunt was performed. The penis is flaccid. (A) Right lateral view of the penis. (B) Ventral view of the penis. (C) Left lateral view of the penis.

At 3 hours post-procedure, significant detumescence was observed, with minimal bleeding from the shunt site (Fig. 2) and a flaccid penile state (Fig. 3). The surgical wound was closed, and the patient was admitted to the intensive care unit (ICU) for close monitoring. Multidisciplinary management with a hemato-oncologist was initiated, and treatment with hydroxyurea and folic acid was started. Investigation by a hemato-oncologist confirmed that the patient had CML based on further examination.

During the first two days of ICU admission, the patient remained hemodynamically stable with no recurrence of priapism. Additional transfusion of three units of PRC was administered. Residual penile discomfort was reported but was tolerable. The patient was subsequently transferred to a general ward.

On the fourth day of hospitalization, the patient showed clinical improvement and was discharged with a hemoglobin level of 9.8 g/dL and a leukocyte count that decreased to 238,480/μL. At a follow-up examination two weeks later, the patient reported no recurrence of priapism and no evidence of erectile dysfunction. Long-term follow-up was scheduled.

## 3 Discussion

Priapism is a urological emergency that occurs in approximately 1.5 cases per 100,000 individuals per year, with hematologic disorders accounting for 20% of cases.^6^^,^^7^ By definition, priapism is an erection lasting 4 hours or more without sexual stimulation.^1^ A thorough history, physical examination, and laboratory tests are required, particularly blood gas analysis, to determine the type of priapism (ischemic or non-ischemic).^4^ This patient's condition was managed according to the clinical findings suggestive of ischemic priapism due to hematologic malignancy, as hematologic disorders are the most common cause of ischemic priapism.^10^

Chronic Myeloid Leukemia is a hematologic malignancy characterized by excessive production of mature granulocytes and typically progresses through chronic, accelerated, and blast phases.^7^ The majority of patients are diagnosed incidentally during the asymptomatic chronic phase.^7^ Priapism as the initial manifestation of CML is extremely rare, occurring in approximately 1–2%.^6^ This condition is thought to be caused by leukostasis and blood clotting, leading to impaired cavernosal outflow.^9^ Contributing factors include tissue hypoxia due to anemia and disruption of the NO–cGMP pathway.^11^

Rapid decision-making regarding emergency urologic management in these cases is crucial.^6^ Ischemic priapism, compared to non-ischemic priapism, requires more rapid treatment to prevent potential cavernosal muscle necrosis.^2^^,^^12^ Functionally, the duration of ischemia is a major determinant of erectile outcome.^12^ Evidence suggests that cavernosal smooth muscle begins to undergo irreversible structural changes after prolonged hypoxia, with a significant increase in erectile dysfunction rates after 24 hours.^12^ In this patient, the onset was approximately 10 hours, which still allows for a better erectile function outcome.^13^

The American Urological Association recommends a stepwise approach to ischemic priapism, beginning with corporal aspiration and intracavernosal injection of phenylephrine.^4^ If detumescence is not achieved, treatment should be escalated immediately to avoid prolonged ischemia and irreversible cavernosal smooth muscle damage.^12^ In these cases, a distal shunt procedure, such as the Ebbehoj technique used in this patient, is recommended as the next-line surgical option.^14^^,^^15^ This technique involves repeated incisions in the glans penis to decompress the corpora cavernosa and achieve rapid detumescence.^15^ This procedure creates a connection between the corpora cavernosa, allowing for the drainage of stagnant, oxygen-depleted blood and the influx of fresh, oxygen-rich blood.^14^^,^^15^

Finally, it's important to remember that this case highlights not only the need for surgery but also a more etiologically focused approach aimed at preventing recurrence.^4^ In this situation, multidisciplinary care, including hemato-oncology, is necessary, given the patient's sexually productive age, to maintain a good quality of life.^1^

## 4 Conclusion

Priapism can be a rare sign of chronic myeloid leukemia and requires immediate, multidisciplinary treatment. Effective management is crucial for achieving detumescence, preventing complications, and preserving erectile function. A comprehensive approach that focuses on the underlying cause is essential to optimize both immediate outcomes and long-term quality of life.

## CRediT authorship contribution statement

**Muhammad Farras Afif Syamhudi:** Writing – original draft, Project administration. **Agung Prasetyo Nitisasmito:** Validation, Supervision, Conceptualization. **Encep Ivan Setiawan:** Validation, Supervision.

## Informed consent

Written informed consent was obtained from the patient and her parents for publication of this case report and accompanying images.

## Approval of the research protocol by an institutional reviewer board

Ethical approval was not required for this case report according to institutional policy.

## Declaration of competing interest

The authors declare no conflict of interest.
